# Total Control – Pollen Presentation and Floral Longevity in Loasaceae (Blazing Star Family) Are Modulated by Light, Temperature and Pollinator Visitation Rates

**DOI:** 10.1371/journal.pone.0041121

**Published:** 2012-08-20

**Authors:** Tilo Henning, Maximilian Weigend

**Affiliations:** 1 Institut für Biologie, Morphologie und Systematik der Phanerogamen, Freie Universität Berlin, Berlin, Germany; 2 Nees-Institut für Biodiversität der Pflanzen, Rheinische Friedrich-Wilhelms-Universität, Bonn, Germany; RIKEN Plant Science Center, Japan

## Abstract

Stamen movements can be understood as a mechanism influencing pollen presentation and increasing outbreeding success of hermaphroditic flowers via optimized male function. In this study we experimentally analyzed the factors regulating autonomous and thigmonastic (triggered by flower visitors) stamen movements in eight species of Loasaceae. Both types of stamen movements are positively influenced by light and temperature and come to a virtual standstill in the dark and at low temperatures (12°C). Pollen presentation is thus discontinued during periods where pollinators are not active. Overall stamen presentation increases with increasing flower age. Contrary to expectation, no geometrical correlation between the floral scale stimulated and the stamen fascicle reacting exists, indicating that the stimulus is transmitted over the receptacle and stamen maturation dictates which and how many stamens react. Thigmonastic stamen presentation is dramatically accelerated compared to autonomous movement (3–37 times), indicating that the rate of stamen maturation can be adjusted to different visitation schedules. Flowers can react relatively uniformly down to stimulation intervals of 10–15 min., consistently presenting comparable numbers of stamens in the flower c. 5 min. after the stimulus and can thus keep the amount of pollen presented relatively constant even under very high visitation frequencies of 4–6 visits/h. Thigmonastic pollen presentation dramatically reduces the overall duration of the staminate phase (to 1/3^rd^ in *Nasa macrothyrsa*). Similarly, the carpellate phase is dramatically reduced after pollination, down to 1 d from 4 d. Overall flower longevity is reduced by more than 2/3^rds^ under high visitation rates (<3 d versus 10 d under visitor exclusion) and depleted and pollinated flowers are rapidly removed from the pool. Complex floral behaviour in Loasaceae thus permits a near-total control over pollen dispensation schedules and floral longevity of the individual flower by an extraordinary fine-tuning to both biotic and abiotic factors.

## Introduction

The lack of obvious movements is one of the most striking features differentiating plants from animals. Plant movements have therefore particularly fascinated scientists across the ages [1],[2]. Some plant movements have been studied in detail, such as the trap mechanisms of *Dionaea muscipula* and *Aldrovanda vesiculosa* [3], or the leaf movements of *Mimosa pudica* [4] and *Albizzia julibrissin* [5]. It has been reported that plant movements are influenced by temperature (in *Dionaea muscipula* and *Aldrovanda vesiculosa*: [3]; *Mimosa pudica*: [4]; *Albizzia julibrissi* [5]), and light (solar tracking) [6], or the lack thereof (nyctinasty, e.g. in *Espeletia schultzii*) [7]. Plant movements can have a range of functions, most frequently serving to protect the plants from damage, but sometimes functioning in trap mechanisms or in pollination ecology. In terms of male function of flowers, stamen movements are the most common type: Thigmonastic stamen movements in *Berberis* (Berberidaceae) were amongst the first active floral movements to be reported in the scientific literature [8], but stamen movements have since also been reported from a range of taxa such as *Sparmannia* (Malvaceae) [9], *Portulaca* (Portulacaceae) [10], *Nigella* (Ranunculaceae) [11] and *Opuntia* (Cactaceae) [12]. In all these cases the movement of the anthers is triggered by a mechanical stimulus and usually leads to the simultaneous outward or inward movement of all stamens present, maximizing pollen deposition on visiting insects. In his review on “rapid plant movements”, Sibaoka [3] ascribes the thigmonastic response of several plant species (*Berberis* spp., *Sparmannia africana*, *Mahonia aequifolium*, *Helianthemum vulgaris*, *Portulaca grandiflora*) to a stimulation of the filaments by flower visitors.

Thigmonastic stamen movements generally concern all stamens of the androecium and are caused by stimulation of the stamen (filament) itself. The response is thus fairly simple and shows little plasticity. Exceptions are found in *Berberis*, where the number of stamens moved depends on the strength of the mechanical stimulus [13] and *Opuntia*, where the direction of the movement depends on the site of the stimulus [14].

**Figure 1:**
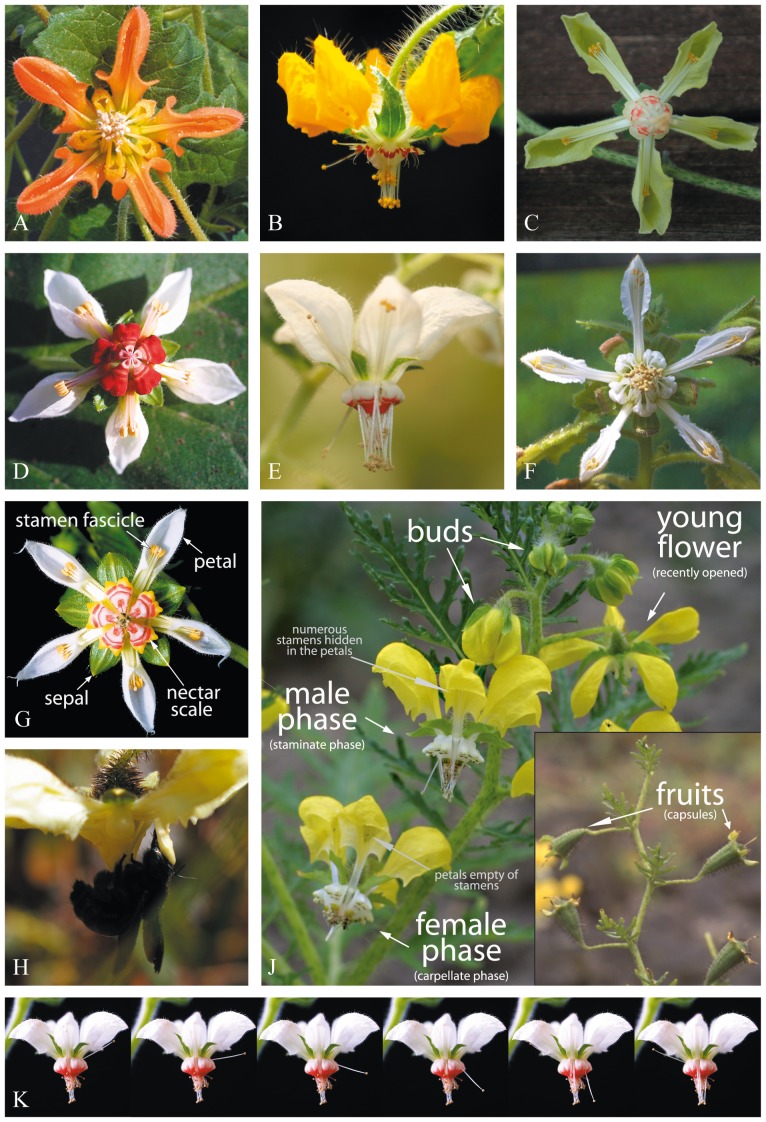
Flowers and floral morphology of species of Loasaceae subfam. Loasoidea used in this study. A. *Caiophora cirsiifolia*, B. *Loasa insons*, C. *Nasa macrothyrsa*, D. *Nasa moroensis*, E. *Nasa vargasii*, F. *Presliophytum heucheraefolium*, G. Flower of *Nasa vargasii*, important floral organs labelled, H. *Neoxylocopa lachnea* [46], harvesting nectar on *Nasa macrothyrsa*, note the outwards bent nectar scale, J. Inflorescence of *Nasa urens* with flowers in different stages of the anthesis, right: fruiting inflorescence, K. Sequence of the stamen movement in *Nasa vargasii*. A mature stamen leaves the boat-shaped petal, passes the gap between two nectar scales and reaches the center of the flower to present its pollen.

The South American Loasaceae subfam. Loasoideae (Fig. 1) show a much more complex type of floral behaviour. The flowers have complex staminodial complexes, so-called nectar scales, into which nectar is secreted. These nectar scales are often contrastingly coloured and their shape and colour correlate to specific pollination syndromes [15]. The polyandric androecium of these plants consists of numerous, initially reflexed stamen bundles of sequentially maturing anthers (Fig. 1B, G, J, K) alternating with the staminodial complexes (Fig. 1G). In the absence of flower visitors, mature anthers present their pollen in the center of the flower by autonomously moving into an upright position (Fig. 1B, K) [16], [17]. Anther dehiscence occurs immediately before or during the stamen movement. At the end of the staminate phase all stamens have moved into the center of the flower and the petals are empty (Fig. 1J). As first reported by Schlindwein & Wittmann [18] the manipulation of the floral scales in *Caiophora arechavaletae* (Urb.) Urb. & Gilg, leads to a direct reaction in the stamen bundles, causing the thigmonastic movement of individual stamens from a reflexed position into the center of the flower. These staminodial complexes are flexible, and are forcibly bent outwards by the flower visitors whilst they are harvesting the nectar (Fig. 1H). Pollen is thereby passively deposited ventrally on the visitors' abdomen or is collected actively by the pollinator [19], [20]. These thigmonastic stamen movements have since been reported from a range of taxa in Loasaceae subfam. Loasoideae (genera *Blumenbachia*, *Caiophora*, *Loasa*, *Nasa*) [18], [19], [20], [21], [22], [23]. Unlike in other flowers, there is spatial separation between the place of manipulation (the floral scale) and the place of the reaction (the stamen bundle). It is unclear how the stimulus is communicated from the floral scale to the anthers that show the thigmonastic reaction and it is also unknown whether the stimulus from an individual staminodial complex is communicated to all five stamen bundles of the flower or affects only the two stamen bundles neighbouring the floral scale that has been manipulated.

The function of thigmonastic anther presentation in Loasaceae has been explained in the framework of the **P**ollen **P**resentation **T**heory (***PPT***) [24], [25], [26], [27]: PPT argues that pollen release in plants is adjusted to the frequency of flowers visits in order to optimize male function. A selection for increased male fitness in hermaphrodite flowers may favor a protracted period of pollen donation with pollen released in minor amounts by dispensing or packaging [28]. This probably increases the likelihood of siring outcrossed offspring. The rapid presentation of fresh pollen in the flower shortly after a flower visit and nectar depletion likely reduces self-pollen deposition and increases pollen export [22]. Stamen movement in Loasaceae subfam. Loasoideae is present in several genera of the group and differs from stamen movements in other plant families: In Loasoideae, individual stamens in the polyandrous androecium are triggered by the manipulation of a separate floral organ (the floral scale) and this mechanism leads to numerous separate stamen movements across the staminate phase, i.e., in the course of several days. Thigmonastic stamen movements are present across pollination modes in Loasoideae in taxa pollinated by short-tongued bees, long-tongued bees and hummingbirds (unpublished observation). Weigend *et al.* [22] therefore detached the floral behaviour from the behaviour of individual pollinators and offer a general functional explanation: Rapid thigmonastic stamen presentation is advantageous, irrespective of pollinator species, ensuring the availability of pollen in flowers recently depleted of nectar and thus makes optimal use of the “hungry pollinators”: Flies, bees and hummingbirds that visit flowers that have been recently visited and where nectar is depleted are more likely to move on to different plants [29],[30], [31], [32], [33] and thus are more likely to sire outcrossed offspring. Rapid, thigmonastic pollen presentation ensures that these valuable “hungry pollinators” are dusted with pollen. Theoretical works on pollen dispensing schedules [27], [34] “predicted” such a phenomon as the “unlikely case in which the number of visits to be received is higly predictable and the individual plant possess the ability to adjust pollen-dispensing schedules accordingly”. If so, they underscored: “plant fitness may increase substantially”. Pollinator behaviour and especially visitation rates may vary throughout the time a flower is presenting pollen. Visitation frequency may change depending on weather conditions, daytime or competition among pollinators. Anther maturation and dehiscence are commonly correlated to temperature, as is overall duration of anthesis. However, there are few publications on a correlation of stamen movements and temperature or light. Only Jaffe *et al.* [35] could prove that the thigmotropic stamen movement of *Portulaca grandiflora* Hook. is positively correlated to the ambient temperature, but not to light intensity. It is currently unknown in how far thigmonastic and autonomous stamen movements, as found in Loasaceae subfam. Loasoideae, are dependent on light or temperature: If autonomous stamen movement was independent of light and temperature, then a large number of mature stamens would be autonomously presented during the night. As pollinator activity follows a diurnal rhythm, the passive presentation of pollen via the autonomous stamen movement as known for Loasoideae [22] would represent a “waste” of pollen during the night. In their natural habitat (mostly the Andes of Southern America), Loasoideae underlie a distinct nightime temperature drop. Pollinator activity is generally limited by thermal constraints, which in turn are (especially in the high Andes) closely correlated to solar radiation [36]. The dramatic fluctuations between day- and nighttime temperatures at high altitudes and the rapid sunset and sunrise close to the equator mainly determine pollinator activity. It would therefore be expected that these abiotic factors affect stamen movement. However, so far it is unclear to which degree flowers of Loasaceae are able to adjust pollen presentation to these different external factors.

Another interesting aspect is the possible effect of thigmonastic stamen presentation on the overall duration of the male phase and flower longevity. Flower longevity is a crucial aspect of pollination biology: Withdrawal of successfully pollinated flowers from the overall flower stock ensures the concentration of pollinator activity on those flowers still requiring their attention [37], [38], [39]. Flower longevity reflects the ecological, genetical and physiological constraints a plant is subject to during flowering [37]. Flower longevity is often considered as being mainly influenced by an either extended (unpollinated) or shortened (successfully pollinated) stigmatic phase. Ashman & Schoen [40] revealed a correlation between the duration of floral attractivity and visitation frequency independent of successful pollination. In Loasoideae, these two factors are likely to be directly linked. Since pollinator frequency (visitation rate) varies [22] and the pollen presentation (staminate phase) within the anthesis is accelerated by the pollinator, it would be expected that the entire staminate phase can be shortened or extended accordingly. This in turn would directly affect flower longevity, since the female phase follows immediately upon the male phase.

There are thus a range of open questions in the context of (autonomous and thigmonastic) stamen movement in Loasaceae, which we want to address in this study:

Do light and temperature affect autonomous and thigmonastic stamen movement?

Can the stamen movement be adjusted to different visiting intervals?

Is the accelerated pollen presentation associated with an accelerated stamen maturation and the overall duration of the staminate phase thus influenced by the number of (simulated) flower visits?

Is the duration of the carpellate phase influenced by pollination?

Does the age of the individual flower (during the staminate phase) influence the number of stamens presented under constant visitation rates?

Is there a spatial relationship between the nectar scale manipulated and the stamen bundle from which an anther/anthers are presented?

## Materials and Methods

### Ethics statement

All necessary permits were obtained for the described field studies. The field studies were undertaken on public land based on the permit “Autorización No. 034-2006-Inrena-IFFS-DCB” issued by the Instituto Nacional de Recursos Naturales in Lima, Peru for T. Henning, G. Brokamp and A. Cano E. The study does not include any endangered or protected species.

### Plant material and cultivation

Observations on the flowers were made on cultivated plants in the greenhouses at the Institut für Biologie, Freie Universität Berlin. Seeds mostly collected on field trips to Peru had been used to raise plants of these species in the greenhouse [vouchers: *Caiophora cirsiifolia*: M. Weigend 7559 (BSB), Fig. 1A; *Loasa insons* Weigend 8724 (BSB), Fig. 1B; *Nasa dyeri* ssp. *australis*: N. Dostert 98/80 (M, USM), Fig. 1G; *Nasa macrothyrsa*: M. Weigend *et al.* 7471 (B, M, USM, HUT), Fig. 1C, H; *Nasa moroensis*: M. Weigend 8424 (BSB, HUT, USM), Fig. 1D; *Nasa urens*: M. Weigend & H. Förther 97/542 (BSB), Fig. 1J; *Nasa vargasii*: M. Weigend 5463 (B, HUT, M, USM), Fig. 1E, K; *Presliophytum heucheraefolium*: M. Weigend 7691 (BSB, USM), Fig. 1F]. Plants were raised and cultivated as described earlier. (For detailed information see: [22], or contact the authors). The number of individuals of the plant species used for this study varied. *N. macrothyrsa* and *P. heucheraefolium* are tall, profusely flowering (>100 flowers) shrubs. Three (*N. macrothyrsa*) and five (*P. heucheraefolium*) tall plants were used for the experiments conducted on these two taxa. The remaining species are smaller, herbaceous plants with a smaller number of flowers per individual. 20 individuals of each of these species were brought into flower, and all plants available were included in the experimental setup.

### Flower longevity and artificial pollination

Flowers of *Nasa* are proterandrous, i.e., the flowers gradually present their stamens with dehisced anthers during early anthesis (staminate phase), after which the stamens wilt and the style elongates and the stigma becomes receptive (carpellate phase) (Fig. 1J). Flowers of *Nasa macrothyrsa* were individually marked and the different phases of the anthesis where noted three times a day. Pollination was carried out by transferring pollen from entire, freshly deshisced anthers of flowers from different plants to the stigmatic surfaces.

### Stamen movement

Stamen movement patterns (autonomous and thigmonastic) were investigated for five species out of three different genera, namely: *Loasa insons, Nasa dyeri* ssp. *australis*, *Nasa macrothyrsa*, *Nasa urens* and *Presliophytum incanum*. Mid-anthetic flowers (e.g. Fig. 1E) were individually marked and mature anthers that already had moved into the center of the flower where cut off one hour prior to the experiment. Stamens that already have presented their pollen in the center of the flower wilt and contract rapidly (Fig. 1J – female phase). The stamen movement from the stamen fascicle inside the petals into the center of the flower is rapid and takes between one to three minutes (Fig. 1K).

### Autonomous stamen movement

Flowers were prepared as described above and kept at different temperatures and light exposures to examine the characteristics and regulatory mechanisms of the autonomous stamen movement. The influence of temperature on stamen movement was investigated based on the temperatures measured in the habitat of *Nasa macrothyrsa*. Autonomous stamen movement overnight (13.5 hours) was investigated at 22°C (day time temperature in the field) and 12°C (night time temperature in the field), respectively. Flowers were kept in the dark and autonomously presented stamens where counted the next morning before sunrise. The data for the autonomous movement during the day at 22°C were calculated from the data of flower longevity (under pollinator exclusion) made at the same time in our greenhouses. Therefore, the total number of stamens per flower was divided by the duration of the staminate phase minus the number of stamens that are expected to be autonomously moved at night (12°C, 13.5 h).

### Thigmonastic stamen movement

All species here studied are bee-pollinated [15]. Bees, irrespective of bee genus, show similar handling behavior – landing on and holding on to the floral scales using footholds provided on the back of the nectar scales (Fig. 1H), quickly probe each nectar scale and thereby insert the proboscis into one floral scale after the other by turning in a circle in the center of the flower. Floral design makes handling of the flower from the periphery impossible. This procedure takes only a few seconds and artificial manipulation thus closely imitated this behavior: Stamen movement was triggered experimentally by slightly bending all five nectar scales outwards with a dissecting needle and thus imitating a pollinator visit (cf. Fig. 1H). Stamens were counted after they reached an upright position. In the experiments at night, the flowers were controlled periodically for stamen movement with a small, dimmed pocket lamp. In all experiments, the stimulus was given in five consecutive intervals irrespective of interval length. Only flowers in the middle staminate phase (see below, “flower age”) were used in this experiment.

### Regular visitor frequency/30 minute interval

The timing of experimental visits to flowers should reflect the natural visitation rate [28]. Accordingly five consecutive 30 minute intervals between stimuli were chosen, based on field observations on *Nasa macrothyrsa* indicating an average interval between two visits to individual flowers of c. 25 minutes [22]. These experiments where carried out on all species.

Five additional stimulation intervals were chosen, imitating different visitor abundancies. For *Nasa macrothyrsa* three datasets (30, 60, 180 min. intervals) are compared to examine low visitation rates. For the 60 min. interval flowers were stimulated (as described above) every hour for 10 consecutive hours. The experiment was carried out one day and the influence on the duration of the staminate phase was calculated from total stamen number. For the very low visitor frequency (180 min. interval) flowers were manipulated three times a day at 9 am, 12 am and 3 pm, respectively, over the entire staminate phase of the flowers. Three other species out of two different genera of Loasaceae were used to investigate the effect of high visitation rates (10, 15, 20 min. intervals). The latter three species [(*Loasa insons*, *Nasa dyeri* ssp. *australis* (*Nasa triphylla* – group, [41]) and *Nasa urens* (*Nasa poissoniana* – group, [42])] are all rather distantly related, entomophilous taxa. They share the same basic flower morphology and pollination system and the data here presented are thus representative for a large proportion of Loasaceae subfam. Loasoideae. The short intervals were tested to investigate to which degree stamen presentation can be accelerated under high visitation rates.

### Location of the response relative to the stimulus

Five different species were examined in this respect, namely: *Nasa moroensis*, *N. macrothyrsa*, *N. vargasii*, *Caiophora cirsiifolia* and *Presliophytum heucheraefolium*. The flowers were prepared as described above. The position of the scales in comparison to the stamen-fascicles was noted on a flower diagram. A single nectar scale was chosen randomly and stimulated for a total of six times at 30 min. intervals. The number of moving stamens reacting from each fascicle was recorded.

### Flower age

Flowers of *Nasa urens* were prepared and manipulation was carried out as above with a 30 min. interval between stimulations. Prior to the experiment, flowers were sorted into four categories (n = 10 flowers each) relative to the stage of the staminate phase. 1. beginning - flowers with≤two stamens already presented; 2. early - 2–10 stamens presented, 3. middle - >10 stamens presented and >10 stamens still reflexed into the petals; 4. late - ≤10 stamens still hidden in the petals.

### Statistics

**Figure 2:**
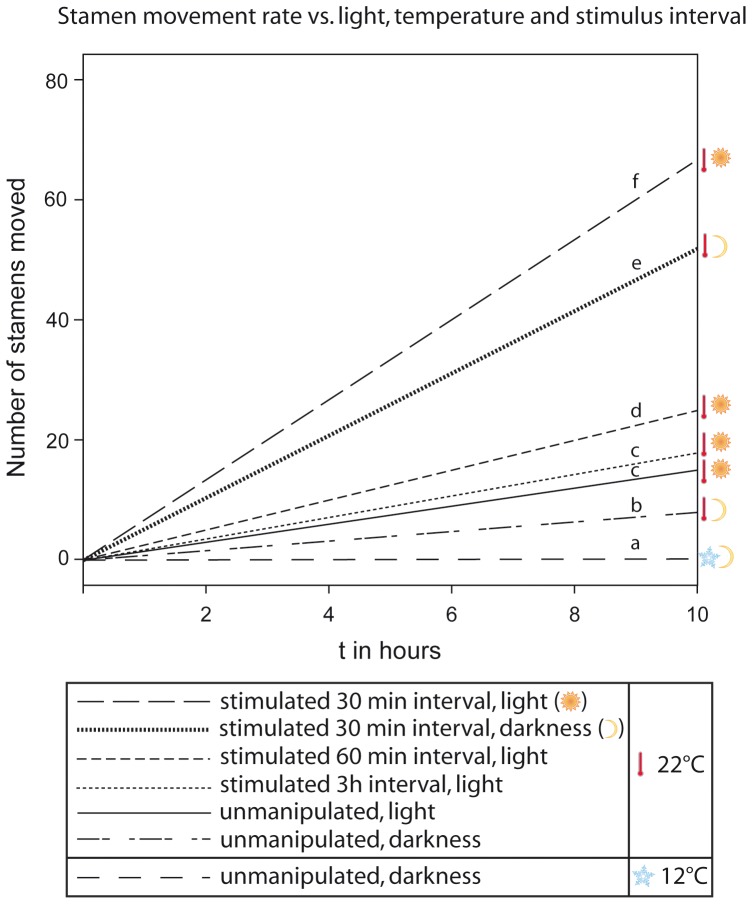
Rate of the stamen movement depending on different abiotic factors and visitation rates in *Nasa macrothyrsa*. Different letters indicate significant differences between the treatments in the number of stamens moved per unit time (gls model; *P*<0.05, Supporting Information S1).

Statistics were done with R (version 2.13.0). To compare the stamen movement of flowers in different staminate phases, a one-factorial ANOVA followed by a Tukey HSD post-hoc test was conducted. Normal distribution and homogeneity of the data were tested by the Shapiro-Wilk Normality Test and the Bartlett Test of Homogeneity of Variances, respectively. In the case of the stamen movement vs. light, temperature and stimulus intervals (dataset *Nasa macrothyrsa*, Tab. 1 Fig. 2) and the datasets on the different visiting intervals (*Nasa urens*, *Loasa insons*, *Nasa dyeri* ssp. *australis* Tab. 2), the underlying assumptions of the ANOVA were not met due to unequal variances. Here, a fitted linear model using generalized least squares using the function “gls” of the package “nlme” [43], including the formula “weights = varIdent(1)” [44], was applied. The R ouput file is added as Supporting Information S1. The data on the location of the response relative to the stimulus was tested for significant differences using SPSS® for Windows®. A Friedmann-test was conducted to reveal overall significant differences in the datasets. If significant differences were found (*P. heucheraefolium*), a Wilcoxon-signed rank test has been used to detect the detailed differences between all stamen bundles.

**Table 1:** Stamen movement depending on stimulation and abiotic factors in *N. macrothyrsa*.

nectarscales stimulated	duration of experiment in hours	stimulus interval in min	light	temperature in °C	n	average stamen-movement per h
yes	2.5	30	daylight	22	20	6.78±1.58
yes	2.5	30	darkness	22	20	5.2±1.27
yes	5	60	daylight	22	20	2.83±0.24
yes	9	180	daylight	22	21	1.83±0.13
no	2.5		daylight	22	42	1.54±0.14
no	13.5		darkness	22	25	0.81±0.31
no	13.5		darkness	12	26	0.025±0.42

**Table 2:** Average stamen movement values per flower at different stimulus intervals (bold: experimentally derived data, rest: calculated).

species	*Loasa insons*					*Nasa dyeri* ssp. *australis*					*Nasa urens*				
stimulus interval in minutes	10	15	20	30	60	10	15	20	30	60	10	15	20	30	60
stamens moved per hour	4.56	4.84	4.74	5.04	3.22	6.78	5.93	5.22	4.87	3.63	5.88	9.84	4.38	4.79	2.39
total amount of stamens moved per stimulus	**0.76**	**1.21**	**1.58**	**2.52**	**3.22**	**1.13**	**1.48**	**1.74**	**2.44**	**3.63**	**0.98**	**2.46**	**1.46**	**2.39**	**2.39**
stimulus response (tot. movement - autonomous movement)	0.49	0.8	1.03	1.7	1.58	0.55	0.61	0.57	0.69	0.13	0.96	2.42	1.41	2.32	2.24
response within first 5 minutes after stimulus	**0.48**	**0.43**	**0.71**	**1**	**0.81**	**0.61**	**0.87**	**0.93**	**1.02**	**0.71**	**0.9**	**2.28**	**0.98**	**1.19**	**1.19**
autonomous stamen movement	0.27	0.41	0.55	**0.82**	1.64	0.58	0.88	1.17	**1.75**	3.5	0.25	0.38	0.5	**0.75**	1.5

## Results

### Autonomous and thigmonastic stamen movement dependent on light and temperature

Fig. 2 compares the autonomous and thigmonastic stamen presentation in flowers of *Nasa macrothyrsa* depending on temperature and light. Treatments and detailed results are summarized in Table 1. Flowers showed an autonomous stamen movement of 1.5 stamens/flower/h in the light at 22°C, but only of 0.8 stamens/flower/h in the dark at 22°C. In the dark and at 12°C, stamen movement almost comes to a standstill with 0.025 stamen/flower/h. Thigmonastic stamen movement was also higher (6.78 st./h) at 22°C in the light than in the dark at the same temperature (5.2 st./h). Autonomous and thigmonastic stamen movement are thus positively correlated to both light and temperature.

### Visitation rates, thigmonastic stamen movement and the duration of the male phase

**Figure 3:**
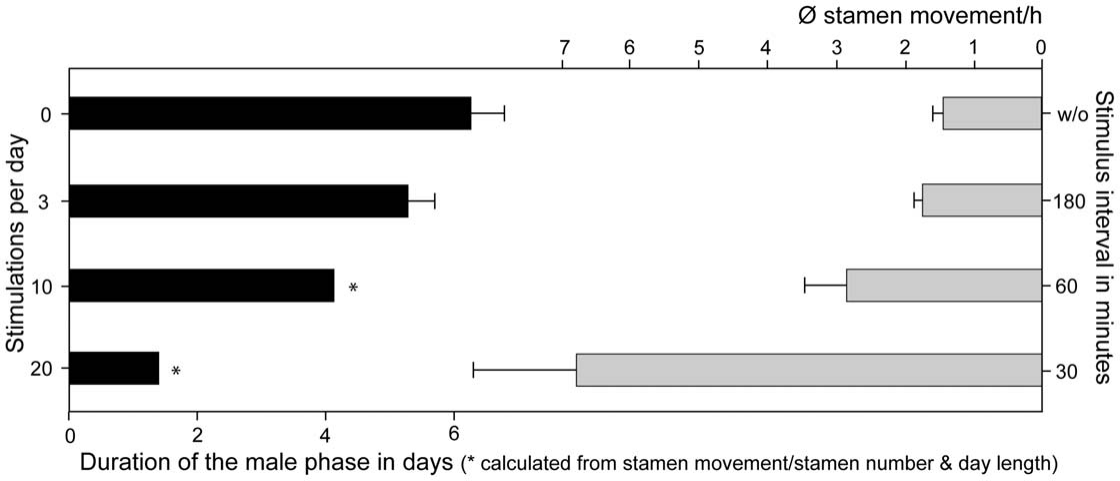
Influence of different visitation rates (stimulus intervals) on the stamen movement rate and the corresponding lenght of the staminate phase in *Nasa macrothyrsa*.

Three different scenarios where tested, imitating different visitation rates (Tab. 1, Fig. 2). Simulated flower visits at 3 h intervals only marginally increased stamen presentation to 1.83±0.13 stamens/h compared to autonomous stamen presentation (1.542±0.141, n = 42). Simulated flower visits at 1 h intervals nearly doubled stamen presentation compared to autonomous movement (to 2.83±0.6 stamens/h). Visitation frequencies observed in the field are close to 30 min. [22]. Imitating these intervals leads to a presentation of 6.78±1.59 stamens/h, i.e., more than four times the rate of autonomous stamen presentation. The flowers of *N. macrothyrsa* have ca. 100 stamens each (98±10, n = 42) and the duration of the staminate phase depends on the number of stamens presented per unit time: Based on stamen presentation rates under different (simulated) flower visitation rates the duration of the staminate phase falls from ca. 6 d (6.255d±0.522, n = 55) in the absence of flower visitors to ca. 5 days (5.275d±0.419, n = 21) with three visits per day, to ca. 4 d (4.122 d, n = 21, calculated from the 60 min.-experiment conducted for 10 h) under hourly visitation. Under visitation frequencies of 30 min., i.e., close to those observed in the field, the staminate phase of the anthesis would be reduced to less than 2 days (ca. 1.45 d, n = 20) (Fig. 3).

**Figure 4:**
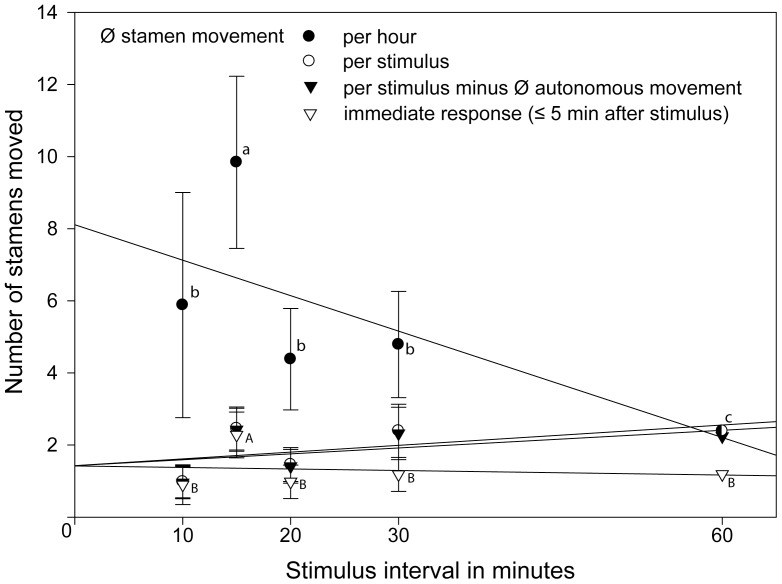
Detailed stamen movement under different visitation rates (stimulus intervals) in *Nasa urens*. Different letters indicate significant differences between the amount of stamens moved (gls model; *P*<0.05, Supporting Information S1).

**Figure 5:**
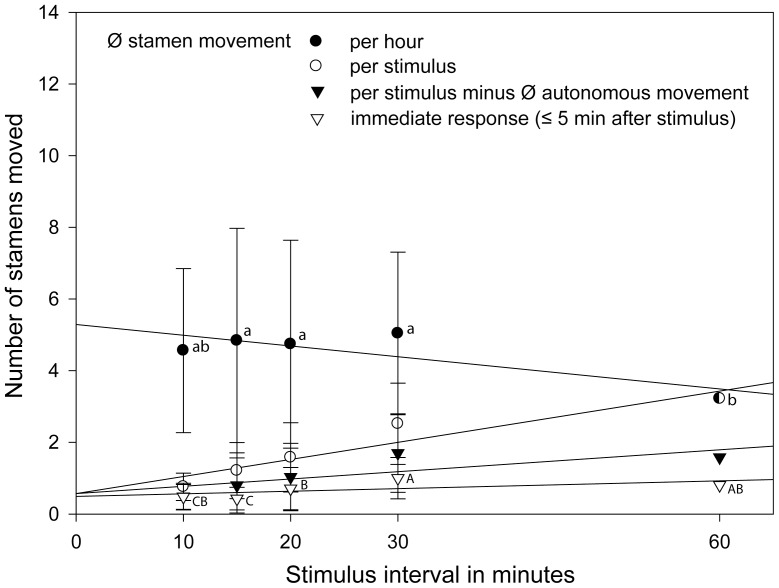
Detailed stamen movement under different visitation rates (stimulus intervals) in *Loasa insons*. Different letters indicate significant differences between the amount of stamens moved (gls model; *P*<0.05, Supporting Information S1).

**Figure 6:**
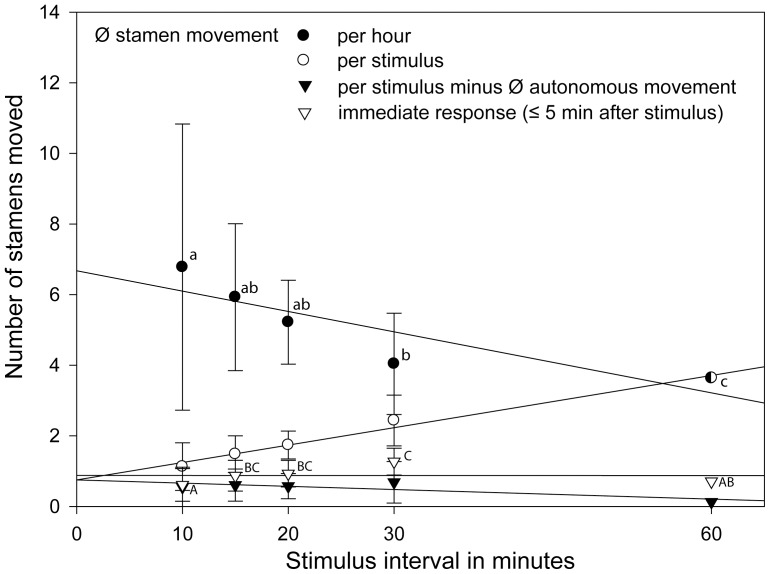
Detailed stamen movement under different visitation rates (stimulus intervals) in *Nasa dyeri* subsp. *australis*. Different letters indicate significant differences between the amount of stamens moved (gls model; *P*<0.05, Supporting Information S1).

Three species were studied with shorter visitation intervals of 10, 15, 20, 30, 60 min. They showed no clear statistically significant differences in the immediate response upon stimulation throughout, i.e., the number of stamens present 5 min. after stimulation is apparently independent of the visitation intervals in all three taxa studied (Tab. 2). *Nasa urens* represents the only exception, with a significantly higher response when stimulated every 15 min (Fig. 4). As would be expected, the total amount of stamens moving per stimulus interval increases with interval length, with the exception of *N. urens*, which peaks at the 15 min. interval. The overall number of stamens presented per hour under different stimulation intervals is variable: In *L. insons* the response shows a slight (not significant) increase from 10 to 30 min., followed by a significant drop to the 60 min. interval (Fig. 5). In *N. dyeri* the shortest interval shows the strongest overall response (stamen movement per hour), which gradually falls with increasing interval length (Fig. 6). In *N. urens* the 10 min. interval shows a strong response, the 15 min. interval the strongest response, which then falls moderately to 20 and 30 min. and again dramatically to 60 min (Fig. 4). A certain proportion of the stamen movements following a stimulus can be ascribed to the autonomous stamen movement that would normally occur in the corresponding time interval. Substracting autonomous movement from the overall movement leads to actual stamen movement caused by stimulation. In both *L. insons* and *N. urens* stimulation at intervals of 60 min. approximately doubles the overall number of stamens moving compared to the autonomous movement (Figs. 4, 5), in *N. dyeri* the stimulus makes no difference and stimulation every 60 min. has no effect on the overall number of stamens moving per unit time (Fig. 6). Conversely, shorter intervals dramatically increase stamen presentation per unit time – by a factor 17 (10 min., *N. dyeri*) respectively 37 (15 min. *N. urens*).

### Thigmonastic stamen movement and flower age

**Figure 7:**
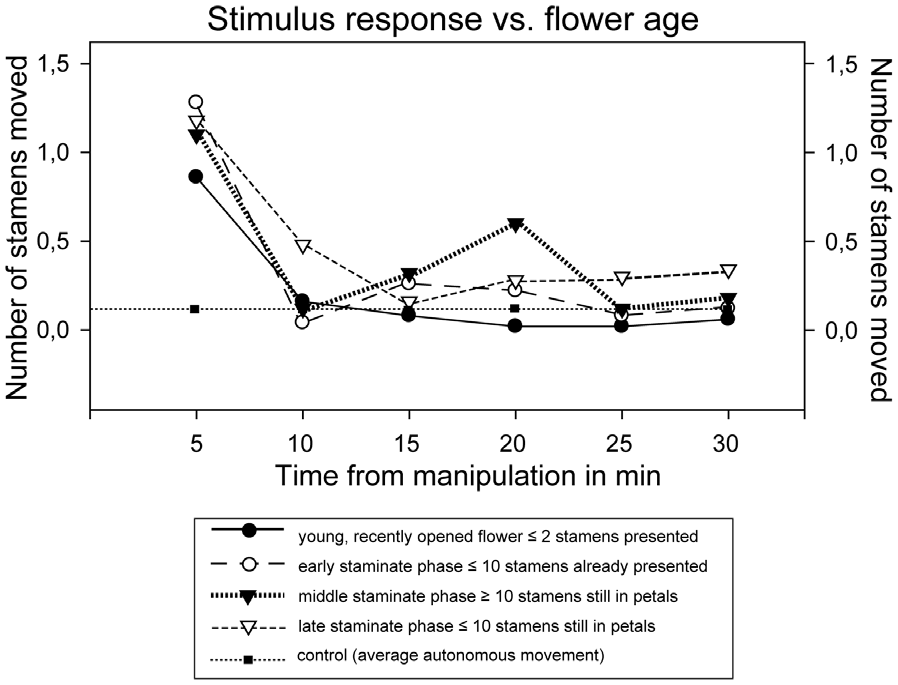
Stamen movement rates in *Nasa urens* per 5 min interval from stimulation at different stages of the anthesis.

**Figure 8:**
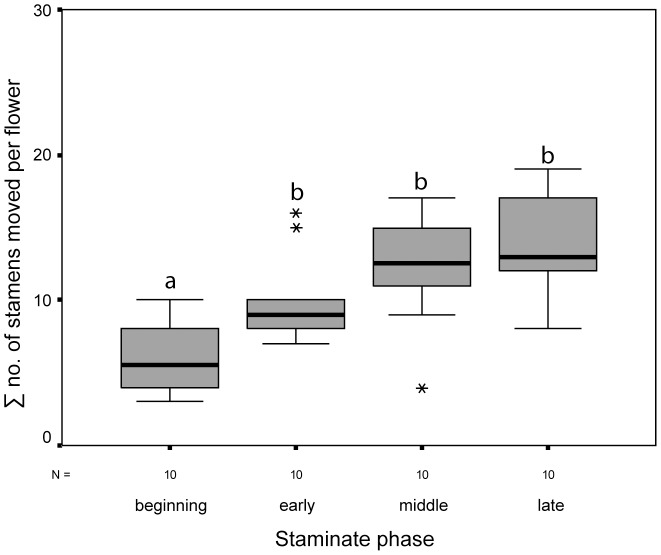
Stem and leaf plot of data for the different flower ages incl. extremes (*) in *Nasa urens*. Different letters indicate significant differences between the amount of stamens moved (gls model; *P*<0.05, Supporting Information S1).

The influence of the “age” of an individual flower on the thigmonastic stamen presentation was tested in *Nasa urens*. Fig. 7 and 8 show the results from the qualitative and quantitative side. The overall number of stamens “triggered” by a stimulus increases with flower age (Tab. 3, Figs. 7, 8): The youngest flowers moved by far the least, the oldest the highest number of stamen in response to a stimulus (Fig. 8). The speed of the response was more or less equal between flower ages, i.e., all flower ages show a rapid reaction within the first five minutes of the stimulus (Tab. 3, Fig. 7). This immediate response is followed by a drop of activity between 10 and 15 minutes after the stimulus. This drop is less pronounced in flowers that are at the very end of the staminate phase. Recently opened flowers show a rather consistent decrease in activity, reaching a minimum between 20 to 25 minutes after the stimulus. All but the youngest flowers show considerable additional stamen movement after the immediate response. Middle-staminate flowers show a second peak of stamen activity in the second half of the time after the stimulus. Flowers at the end of the staminate phase have an even course of stamen activity throughout the period of observation with a weaker drop after the immediate response and a uniformly high activity in the second half of interval. These data indicate that during the course of the staminate phase the rate of stamen maturation gradually increases.

**Table 3:** Thigmonastic stamen movement at different points of the staminate phase.

stamens moved	status of the staminate phase			
	beginning	early	middle	late
Ø within the 1^st^ 5 minutes	0.86	1.28	1.1	1.18
Ø per interval per flower	1.2	2	2.44	2.74
Ø in total (5 intervals) per flower	6	10	12.2	13.7

### Pollination and duration of the carpellate phase

Under pollinator exclusion, the carpellate phase of *Nasa macrothyrsa* has a mean duration of >4 days (4.1 d±0.7, n = 26). Under hand pollination flowers wilt rapidly and the corolla and androecium are shed. The mean duration of the carpellate phase is reduced to ca. 1 day (1.1 d±0.3, n = 27) after hand pollination. Thus, the carpellate phase is dramatically reduced under pollination and is terminated soon after a single successful pollination event.

### Spatial relationship between stimulus and thigmonastic response

**Figure 9:**
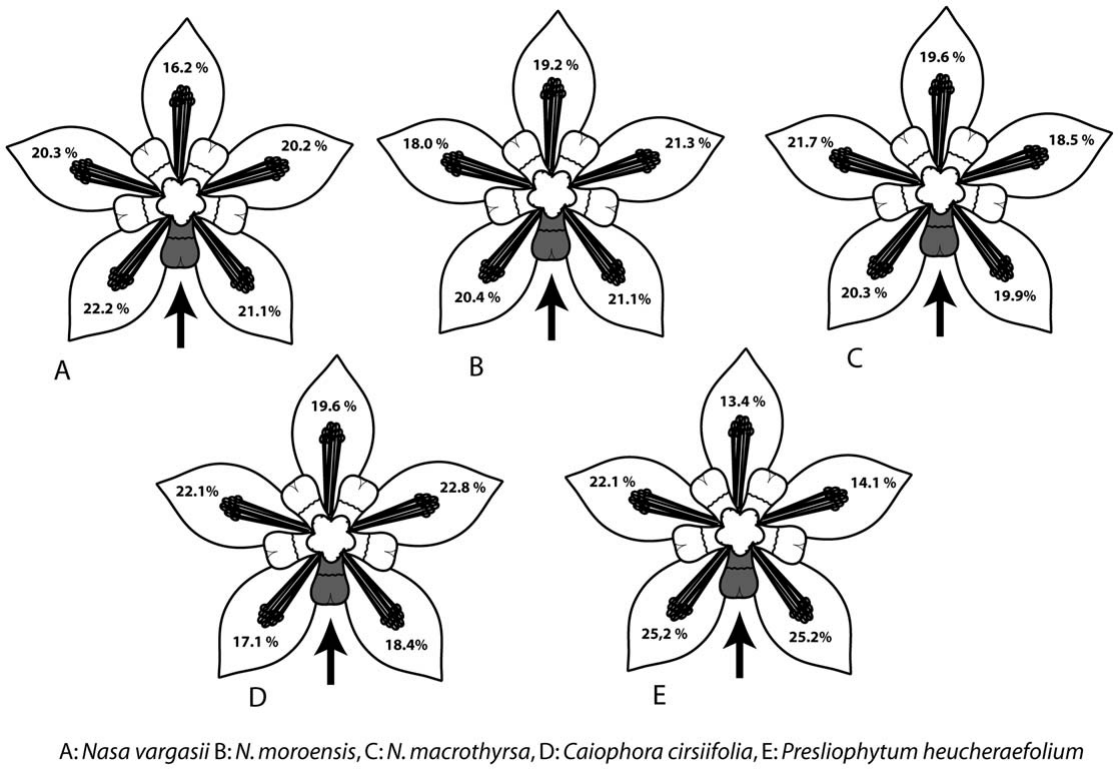
Summary of the data on the stamen origin experimentally obtained. Grey filling/arrow = stimulated nectar scale. Stamen movements from individual stamen fascicles were summed up for five stimulations in 30 Min. intervals. Percentage of stamen movement for each fascicle is given in the corresponding “petal”. A. *Nasa vargasii*, B. *N. moroensis*, C. *N. macrothyrsa*, D. *Caiophora cirsiifolia*, E. *Presliophytum heucheraefolium*.

Five species were examined in order to identify a possible spatial relationship between the trigger (i.e., the nectar scale manipulated) and the responding stamen(-s): *Nasa vargasii* (n = 51 flowers), *N. moroensis* (n = 56), *N. macrothyrsa* (n = 25), *Caiophora cirsiifolia* (n = 23) and *Presliophytum heucheraefolium* (n = 18). If all stamen fascicles reacted equally to the manipulation of a single nectar scale, irrespective of their spatial relationship to the scale, then it would be expected that 20% of the overall thigmonastic stamens would originate from each of the five stamen bundles. Observed summary data were close to this expectation: In *N. vargasii* and *N. moroensis* 19 to 22% of the responding stamens originated in the fascicles directly flanking the manipulated nectar scale (Fig. 9A–C). In *Caiophora cirsiifolia* these fascicles showed a weaker response (17–18%, Fig. 9D). Conversely, in *P. heucheraefolium* these fascicles were the most active (both 25%, Fig. 9E). However, while there are differences in the overall number of thigmonastic stamens from the different fascicles, a statistical comparison (Friedmann-Test) revealed no siginificant differences in any of the species of *Nasa* and *Caiophora* examined (Tab. 4), i.e., the response of the thigmonastic stamens was independent of which nectar scale is stimulated. Only *Presliophytum* seems to have a spatialized stamen reaction (*P*<0.05). A Wilcoxon-Test revealed that mainly those two fascicles neighbouring the manipulated scale differ significantly from the others. It has to be assumed, that these differences can only be found between the two neighbouring and two of the fascicles more distant to the stimulus. The activity of the third remote fascicle varies to a lesser extent. Thus there is no significant difference in the activity of this particular fascicle, compared to the fascicles flank to the scale manipulated. Furthermore, for *P. heucheraefolium* only 18 flowers where available and it cannot be excluded that this significance is an artefact of the smaller sample size.

**Table 4:** Sample size and *P*-values of the experiment on the stamen origin.

taxon	n plants	n flowers	∑ stamen moved	*P*
*Nasa vargasii*	20	51	693	0.201
*N. moroensis*	20	56	573	0.244
*N. macrothyrsa*	3	25	281	0.947
*Caiophora cirsiifolia*	20	23	158	0.569
*Presliophytum heucheraefolium*	5	18	262	**0.011**

## Discussion

The present study on pollen presentation in Loasaceae documents a near total control of the flower over pollen dispensation. Surprisingly, there appears to be no direct spatial correlation between the stamen bundle providing the thigmonastic stamen and the localization of the stimulus. The stimulus is apparently transmitted to all stamen fascicles via the receptacle, leading to a reaction in a determined number of stamens from all fascicles.

The data presented here for a number of selected species demonstrate that the pollen dispensation schedule is modulated by both biotic and abiotic factors: It comes to a virtual standstill in the dark and at low temperatures (here 12°C), so that in the absence of pollinators during the (Andean) nights nearly no pollen is presented. At higher temperatures (here 22°C) and daylight, pollen is gradually presented by autonomous stamen movement, but this stamen movement is slow, leading to a total duration of the staminate phase in *N. macrothyrsa* of ca. 6 days. Under flower visitation the anther presentation schedule can be dramatically accelerated, reducing the staminate phase to less than 2 d, i.e., less than a third, under flower visitation rates of 2 visits/h. Experiments with other species further show that an acceleration of pollen presentation is possible down to visitation intervals of 4 visits/h (in *N. urens*) respectively 6 visits/h (*N. dyeri*). In the field, visitation rates are unlikely to be equal throughout anthesis and differ for each individual flower: The data provided by Weigend *et al.* [22] show a wide spread in visitation intervals. Thus, temperature, light (day/night) and visitation rates fluctuate widely during anthesis and for each individual flower. The direct response of thigmonastic stamen movement to both stimulation and abiotic factors permits an extreme degree of short-term fine-tuning of the pollen presentation schedule and even of the overall duration of the staminate phase, approaching the ideal condition formulated by Harder & Wilson [28]. Stamen presentation per unit time can be dramatically accelerated (up to 37× compared to autonomous movement) and the duration of the staminate phase can be dramatically reduced under very high visitation frequencies. Possibly the most striking result is, that the average number of mature stamens presented in the center of the flower after 5 min. is held virtually constant irrespective of visitation frequencies. Shortly after nectar and pollen depletion any visitor will therefore always find approximately the same amount of pollen (and no nectar) in the center of the flower. Pollen dispensation is thus remarkably even and the amount of pollen presented shortly after a previous visits is more or less equal, irrespective of visitation intervals. Withdrawal of successfully pollinated flowers from the overall flower stock [37], [38], [39] is taken care of very effectively, with flowers terminating their carpellate phase virtually immediately after pollination, whereas unpollinated flowers remain open for a much longer time. Under ideal conditions floral longevity in *N. macrothyrsa* can thus be reduced from over 10 d (>6.25 d staminate, >4.1 d carpellate) to less than 3 d (1.45 d staminate, 1.1 d carpellate), i.e., by more than 2/3rds. Direct observational data for *Nasa macrothyrsa* confirm this conclusion and it can even be extrapolated on shorter intervals. The effect may be even more pronounced in other species with an even more extreme acceleration of stamen presentation: In our experiments we found no saturation in intervals of 30 min. and shorter (at least in *N. dyeri* and in *L. insons*) – the total amount of anthers presented per hour either increased with shorter intervals, or was kept constant. This indicates that even under consistently high pollinator activity (6 visits/h) stamen maturation and presentation can be accelerated sufficiently to ensure the immediate presentation of fresh pollen after a flower visit. The only major difference in stamen presentation is introduced by flower age, with the rate of stamen maturation apparently accelerated in older flowers and consequently overall pollen dispensation gradually increasing throughout the staminate phase. Nevertheless, differences in the amount of stamens moved upon a stimulus at different flower ages are minor compared to the differences caused by changes in visitation rates.

Although not specifically examined here, short pollen viability in general is thought to be linked to pollen allocation modes with a frequent release of small quantities of pollen and vice versa [45]. In the species here studied anthers dehisce immediately before or during stamen movement, so that pollen presented is always fresh and viable. The immature anthers are reflexed and hidden in the petals, so that pollen collecting bees will usually not be able to deplete (“rob”) the pollen of an individual flower in one visit.

Harder & Wilson [28] mentioned the “unlikely case in which the number of visits to be received is higly predictable and the individual plant possesses the ability to adjust pollen-dispensing schedules accordingly” and argued that this would mean that “plant fitness may increase substantially”. The data here presented on several species of Loasaceae provide an unexpectedly extreme example of such total control over pollen-dispensation in flowering plants.

## Supporting Information

Supporting Information S1 **Detailed output of the statistics computed in R.**
(DOC)
