# Supplementary material for: Total Control – Pollen Presentation and Floral Longevity in Loasaceae (Blazing Star Family) Are Modulated by Light, Temperature and Pollinator Visitation Rates
Source: PLoS One. 2012 Aug 20;7(8):e41121. doi: 10.1371/journal.pone.0041121 (PMC3423403; doi:10.1371/journal.pone.0041121)
Supplement: Supporting Information S1 — Detailed output of the statistics computed in R. (DOC) [file pone.0041121.s001.doc]

S1. Statistical results are given here, showing the output computed in R 2.13.0.

Stem and leaf plot of data for the different flower ages incl. extremes (*) in *Nasa urens*:

TukeyHSD(aov)

Fit: aov(formula = stamina ~ phase, data = alter)

$phase

diff lwr upr p adj

early-beginning 4.0 0.09869211 7.901308 0.0427096

late-beginning 7.7 3.79869211 11.601308 0.0000328

middle-beginning 6.2 2.29869211 10.101308 0.0007328

late-early 3.7 -0.20130789 7.601308 0.0683344

middle-early 2.2 -1.70130789 6.101308 0.4371714

middle-late -1.5 -5.40130789 2.401308 0.7299403

Comparison of the actual rate of the stamen movement depending on different abiotic factors and visitation rates in *Nasa macrothyrsa*:

"unmanipulated, darkness, 12°C" as Intercept:

summary(gls)

Coefficients:

Value Std.Error t-value p-value

(Intercept) 0.026667 0.1961676 0.135938 0.8920

stimulusdark22g 0.782222 0.2774229 2.819602 0.0053

stimulusdark30min 5.173333 0.2942515 17.581335 0.0000

stimuluslight180min 1.802527 0.2942515 6.125806 0.0000

stimuluslight22g 1.515910 0.2365871 6.407409 0.0000

stimuluslight30min 6.753333 0.2942515 22.950892 0.0000

stimuluslight60min 2.709958 0.2833897 9.562656 0.0000

"unmanipulated, darkness, 22°C" as Intercept:

summary(gls)

Coefficients:

Value Std.Error t-value p-value

(Intercept) 0.808889 0.1961676 4.123457 0.0001

stimulusdark12g -0.782222 0.2774229 -2.819602 0.0053

stimulusdark30min 4.391111 0.2942515 14.922989 0.0000

stimuluslight180min 1.020305 0.2942515 3.467460 0.0007

stimuluslight22g 0.733688 0.2365871 3.101133 0.0022

stimuluslight30min 5.971111 0.2942515 20.292546 0.0000

stimuluslight60min 1.927736 0.2833897 6.802421 0.0000

"unmanipulated, light, 22°C" as Intercept:

summary(gls)

Coefficients:

Value Std.Error t-value p-value

(Intercept) 1.542577 0.1322562 11.663551 0.0000

stimulusdark12g -1.515910 0.2365871 -6.407409 0.0000

stimulusdark22g -0.733688 0.2365871 -3.101133 0.0022

stimulusdark30min 3.657423 0.2561130 14.280505 0.0000

stimuluslight180min 0.286617 0.2561130 1.119104 0.2646

stimuluslight30min 5.237423 0.2561130 20.449656 0.0000

stimuluslight60min 1.194048 0.2435563 4.902554 0.0000

"stimulated 30min interval, light, 22°C" as Intercept:

summary(gls)

Coefficients:

Value Std.Error t-value p-value

(Intercept) 6.780000 0.2193221 30.913440 0

stimulusdark12g -6.753333 0.2942515 -22.950892 0

stimulusdark22g -5.971111 0.2942515 -20.292546 0

stimulusdark30min -1.580000 0.3101683 -5.094009 0

stimuluslight180min -4.950806 0.3101683 -15.961678 0

stimuluslight22g -5.237423 0.2561130 -20.449656 0

stimuluslight60min -4.043375 0.2998836 -13.483150 0

"stimulated 30min interval, darkness, 22°C" as Intercept:

summary(gls)

Coefficients:

Value Std.Error t-value p-value

(Intercept) 5.200000 0.2193221 23.709423 0

stimulusdark12g -5.173333 0.2942515 -17.581335 0

stimulusdark22g -4.391111 0.2942515 -14.922989 0

stimuluslight180min -3.370806 0.3101683 -10.867669 0

stimuluslight22g -3.657423 0.2561130 -14.280505 0

stimuluslight30min 1.580000 0.3101683 5.094009 0

stimuluslight60min -2.463375 0.2998836 -8.214438 0

"stimulated 60min interval, light, 22°C" as Intercept:

summary(gls)

Coefficients:

Value Std.Error t-value p-value

(Intercept) 2.736625 0.2045189 13.380792 0.0000

stimulusdark12g -2.709958 0.2833897 -9.562656 0.0000

stimulusdark22g -1.927736 0.2833897 -6.802421 0.0000

stimulusdark30min 2.463375 0.2998836 8.214438 0.0000

stimuluslight180min -0.907431 0.2998836 -3.025944 0.0028

stimuluslight22g -1.194048 0.2435563 -4.902554 0.0000

stimuluslight30min 4.043375 0.2998836 13.483150 0.0000

"stimulated 3h interval, light, 22°C" as Intercept:

summary(gls)

Coefficients:

Value Std.Error t-value p-value

(Intercept) 1.829194 0.2193221 8.340218 0.0000

stimulusdark12g -1.802527 0.2942515 -6.125806 0.0000

stimulusdark22g -1.020305 0.2942515 -3.467460 0.0007

stimulusdark30min 3.370806 0.3101683 10.867669 0.0000

stimuluslight22g -0.286617 0.2561130 -1.119104 0.2646

stimuluslight30min 4.950806 0.3101683 15.961678 0.0000

stimuluslight60min 0.907431 0.2998836 3.025944 0.0028

Detailed stamen movement under different visitation rates (stimulus intervals) in *Nasa urens*:

(1) Number of stamens moved per hour:

"10 minutes stimulus interval" as Intercept:

summary(gls)

Coefficients:

Value Std.Error t-value p-value

(Intercept) 5.880000 0.5387772 10.913602 0.0000

stimulus15 3.960000 0.7619460 5.197218 0.0000

stimulus20 -1.500000 0.7619460 -1.968643 0.0527

stimulus30 -1.093333 0.6221263 -1.757414 0.0829

stimulus60 -3.490000 0.6598646 -5.288964 0.0000

"15 minutes stimulus interval" as Intercept:

summary(gls)

Coefficients:

Value Std.Error t-value p-value

(Intercept) 9.840000 0.5387772 18.263579 0

stimulus10 -3.960000 0.7619460 -5.197218 0

stimulus20 -5.460000 0.7619460 -7.165862 0

stimulus30 -5.053333 0.6221263 -8.122680 0

stimulus60 -7.450000 0.6598646 -11.290194 0

"20 minutes stimulus interval" as Intercept:

summary(gls)

Coefficients:

Value Std.Error t-value p-value

(Intercept) 4.380000 0.5387772 8.129520 0.0000

stimulus10 1.500000 0.7619460 1.968643 0.0527

stimulus15 5.460000 0.7619460 7.165862 0.0000

stimulus30 0.406667 0.6221263 0.653672 0.5153

stimulus60 -1.990000 0.6598646 -3.015770 0.0035

"30 minutes stimulus interval" as Intercept:

summary(gls)

Coefficients:

Value Std.Error t-value p-value

(Intercept) 4.786667 0.3110632 15.388085 0.0000

stimulus10 1.093333 0.6221263 1.757414 0.0829

stimulus15 5.053333 0.6221263 8.122680 0.0000

stimulus20 -0.406667 0.6221263 -0.653672 0.5153

stimulus60 -2.396667 0.4918341 -4.872917 0.0000

"60 minutes stimulus interval" as Intercept:

summary(gls)

Coefficients:

Value Std.Error t-value p-value

(Intercept) 2.390000 0.3809730 6.273410 0.0000

stimulus10 3.490000 0.6598646 5.288964 0.0000

stimulus15 7.450000 0.6598646 11.290194 0.0000

stimulus20 1.990000 0.6598646 3.015770 0.0035

stimulus30 2.396667 0.4918341 4.872917 0.0000

(2) Number of stamen moved as immediate response (<5 min after stimulus)

"10 minutes stimulus interval" as Intercept:

summary(gls)

Coefficients:

Value Std.Error t-value p-value

(Intercept) 4.500000 0.6963396 6.462364 0.0000

stimulus15 6.900000 0.9847730 7.006691 0.0000

stimulus20 0.400000 0.9847730 0.406185 0.6858

stimulus30 1.433333 0.8040638 1.782612 0.0787

stimulus60 1.450000 0.8528384 1.700205 0.0932

"15 minutes stimulus interval" as Intercept:

summary(gls)

Coefficients:

Value Std.Error t-value p-value

(Intercept) 11.400000 0.6963396 16.371321 0

stimulus10 -6.900000 0.9847730 -7.006691 0

stimulus20 -6.500000 0.9847730 -6.600506 0

stimulus30 -5.466667 0.8040638 -6.798798 0

stimulus60 -5.450000 0.8528384 -6.390425 0

"60 minutes stimulus interval" as Intercept:

summary(gls)

Coefficients:

Value Std.Error t-value p-value

(Intercept) 5.950000 0.4923865 12.084004 0.0000

stimulus10 -1.450000 0.8528384 -1.700205 0.0932

stimulus15 5.450000 0.8528384 6.390425 0.0000

stimulus20 -1.050000 0.8528384 -1.231183 0.2221

stimulus30 -0.016667 0.6356682 -0.026219 0.9792

Detailed stamen movement under different visitation rates (stimulus intervals) in *Loasa insons*.

(1) Number of stamens moved per hour:

"10 minutes stimulus interval" as Intercept:

summary(gls)

Coefficients:

Value Std.Error t-value p-value

(Intercept) 4.560000 0.5347661 8.527093 0.0000

stimulus15 0.280000 0.7562734 0.370236 0.7120

stimulus20 0.180000 0.7562734 0.238009 0.8124

stimulus30 0.480000 0.7562734 0.634691 0.5272

stimulus60 -1.338947 0.7562734 -1.770454 0.0799

"15 minutes stimulus interval" as Intercept:

summary(gls)

Coefficients:

Value Std.Error t-value p-value

(Intercept) 4.840000 0.5347661 9.050687 0.0000

stimulus10 -0.280000 0.7562734 -0.370236 0.7120

stimulus20 -0.100000 0.7562734 -0.132227 0.8951

stimulus30 0.200000 0.7562734 0.264455 0.7920

stimulus60 -1.618947 0.7562734 -2.140691 0.0349

"60 minutes stimulus interval" as Intercept:

summary(gls)

Coefficients:

Value Std.Error t-value p-value

(Intercept) 3.221053 0.5347661 6.023293 0.0000

stimulus10 1.338947 0.7562734 1.770454 0.0799

stimulus15 1.618947 0.7562734 2.140691 0.0349

stimulus20 1.518947 0.7562734 2.008463 0.0474

stimulus30 1.818947 0.7562734 2.405145 0.0181

(2) Number of stamen moved as immediate response (<5 min after stimulus)

"10 minutes stimulus interval" as Intercept:

summary(gls)

Coefficients:

Value Std.Error t-value p-value

(Intercept) 2.400000 0.4736754 5.066760 0.0000

stimulus15 -0.250000 0.6698782 -0.373202 0.7098

stimulus20 1.150000 0.6698782 1.716730 0.0893

stimulus30 2.600000 0.6698782 3.881302 0.0002

stimulus60 1.652632 0.6698782 2.467063 0.0154

"15 minutes stimulus interval" as Intercept:

summary(gls)

Coefficients:

Value Std.Error t-value p-value

(Intercept) 2.150000 0.4736754 4.538973 0.0000

stimulus10 0.250000 0.6698782 0.373202 0.7098

stimulus20 1.400000 0.6698782 2.089932 0.0393

stimulus30 2.850000 0.6698782 4.254505 0.0000

stimulus60 1.902632 0.6698782 2.840265 0.0055

"20 minutes stimulus interval" as Intercept:

summary(gls)

Coefficients:

Value Std.Error t-value p-value

(Intercept) 3.550000 0.4736754 7.494583 0.0000

stimulus10 -1.150000 0.6698782 -1.716730 0.0893

stimulus15 -1.400000 0.6698782 -2.089932 0.0393

stimulus30 1.450000 0.6698782 2.164573 0.0329

stimulus60 0.502632 0.6698782 0.750333 0.4549

"30 minutes stimulus interval" as Intercept:

summary(gls)

Coefficients:

Value Std.Error t-value p-value

(Intercept) 5.000000 0.4736754 10.555751 0.0000

stimulus10 -2.600000 0.6698782 -3.881302 0.0002

stimulus15 -2.850000 0.6698782 -4.254505 0.0000

stimulus20 -1.450000 0.6698782 -2.164573 0.0329

stimulus60 -0.947368 0.6698782 -1.414240 0.1606

"60 minutes stimulus interval" as Intercept:

summary(gls)

Coefficients:

Value Std.Error t-value p-value

(Intercept) 4.052632 0.4736754 8.555714 0.0000

stimulus10 -1.652632 0.6698782 -2.467063 0.0154

stimulus15 -1.902632 0.6698782 -2.840265 0.0055

stimulus20 -0.502632 0.6698782 -0.750333 0.4549

stimulus30 0.947368 0.6698782 1.414240 0.1606

Detailed stamen movement under diff erent visitation rates (stimulus intervals) in *Nasa dyeri* subsp. *australis*

(1) Number of stamens moved per hour:

"10 minutes stimulus interval" as Intercept:

summary(gls)

Coefficients:

Value Std.Error t-value p-value

(Intercept) 6.780000 0.4730674 14.331996 0.0000

stimulus15 -0.852727 0.6536365 -1.304589 0.1949

stimulus20 -1.560000 0.6690183 -2.331775 0.0216

stimulus30 -1.908571 0.6193906 -3.081370 0.0026

stimulus60 -3.150000 0.6690183 -4.708391 0.0000

"15 minutes stimulus interval" as Intercept:

summary(gls)

Coefficients:

Value Std.Error t-value p-value

(Intercept) 5.927273 0.4510521 13.140995 0.0000

stimulus10 0.852727 0.6536365 1.304589 0.1949

stimulus20 -0.707273 0.6536365 -1.082058 0.2817

stimulus30 -1.055844 0.6027437 -1.751730 0.0827

stimulus60 -2.297273 0.6536365 -3.514603 0.0007

"20 minutes stimulus interval" as Intercept:

summary(gls)

Coefficients:

Value Std.Error t-value p-value

(Intercept) 5.220000 0.4730674 11.034369 0.0000

stimulus10 1.560000 0.6690183 2.331775 0.0216

stimulus15 0.707273 0.6536365 1.082058 0.2817

stimulus30 -0.348571 0.6193906 -0.562765 0.5748

stimulus60 -1.590000 0.6690183 -2.376617 0.0193

"30 minutes stimulus interval" as Intercept:

summary(gls)

Coefficients:

Value Std.Error t-value p-value

(Intercept) 4.871429 0.3998149 12.184209 0.0000

stimulus10 1.908571 0.6193906 3.081370 0.0026

stimulus15 1.055844 0.6027437 1.751730 0.0827

stimulus20 0.348571 0.6193906 0.562765 0.5748

stimulus60 -1.241429 0.6193906 -2.004274 0.0476

"60 minutes stimulus interval" as Intercept:

summary(gls)

Coefficients:

Value Std.Error t-value p-value

(Intercept) 3.630000 0.4730674 7.673325 0.0000

stimulus10 3.150000 0.6690183 4.708391 0.0000

stimulus15 2.297273 0.6536365 3.514603 0.0007

stimulus20 1.590000 0.6690183 2.376617 0.0193

stimulus30 1.241429 0.6193906 2.004274 0.0476

(2) Number of stamen moved as immediate response (<5 min after stimulus)

"10 minutes stimulus interval" as Intercept:

summary(gls)

Coefficients:

Value Std.Error t-value p-value

(Intercept) 3.050000 0.4294346 7.102363 0.0000

stimulus15 1.313636 0.5933491 2.213935 0.0290

stimulus20 1.600000 0.6073122 2.634559 0.0097

stimulus30 2.057143 0.5622618 3.658692 0.0004

stimulus60 0.500000 0.6073122 0.823300 0.4122

"15 minutes stimulus interval" as Intercept:

summary(gls)

Coefficients:

Value Std.Error t-value p-value

(Intercept) 4.363636 0.4094498 10.657318 0.0000

stimulus10 -1.313636 0.5933491 -2.213935 0.0290

stimulus20 0.286364 0.5933491 0.482623 0.6304

stimulus30 0.743506 0.5471503 1.358871 0.1771

stimulus60 -0.813636 0.5933491 -1.371261 0.1732

"20 minutes stimulus interval" as Intercept:

summary(gls)

Coefficients:

Value Std.Error t-value p-value

(Intercept) 4.650000 0.4294346 10.828192 0.0000

stimulus10 -1.600000 0.6073122 -2.634559 0.0097

stimulus15 -0.286364 0.5933491 -0.482623 0.6304

stimulus30 0.457143 0.5622618 0.813043 0.4180

stimulus60 -1.100000 0.6073122 -1.811260 0.0730

"30 minutes stimulus interval" as Intercept:

summary(gls)

Coefficients:

Value Std.Error t-value p-value

(Intercept) 5.107143 0.3629384 14.071650 0.0000

stimulus10 -2.057143 0.5622618 -3.658692 0.0004

stimulus15 -0.743506 0.5471503 -1.358871 0.1771

stimulus20 -0.457143 0.5622618 -0.813043 0.4180

stimulus60 -1.557143 0.5622618 -2.769427 0.0066

"60 minutes stimulus interval" as Intercept:

summary(gls)

Coefficients:

Value Std.Error t-value p-value

(Intercept) 3.550000 0.4294346 8.266685 0.0000

stimulus10 -0.500000 0.6073122 -0.823300 0.4122

stimulus15 0.813636 0.5933491 1.371261 0.1732

stimulus20 1.100000 0.6073122 1.811260 0.0730

stimulus30 1.557143 0.5622618 2.769427 0.0066
